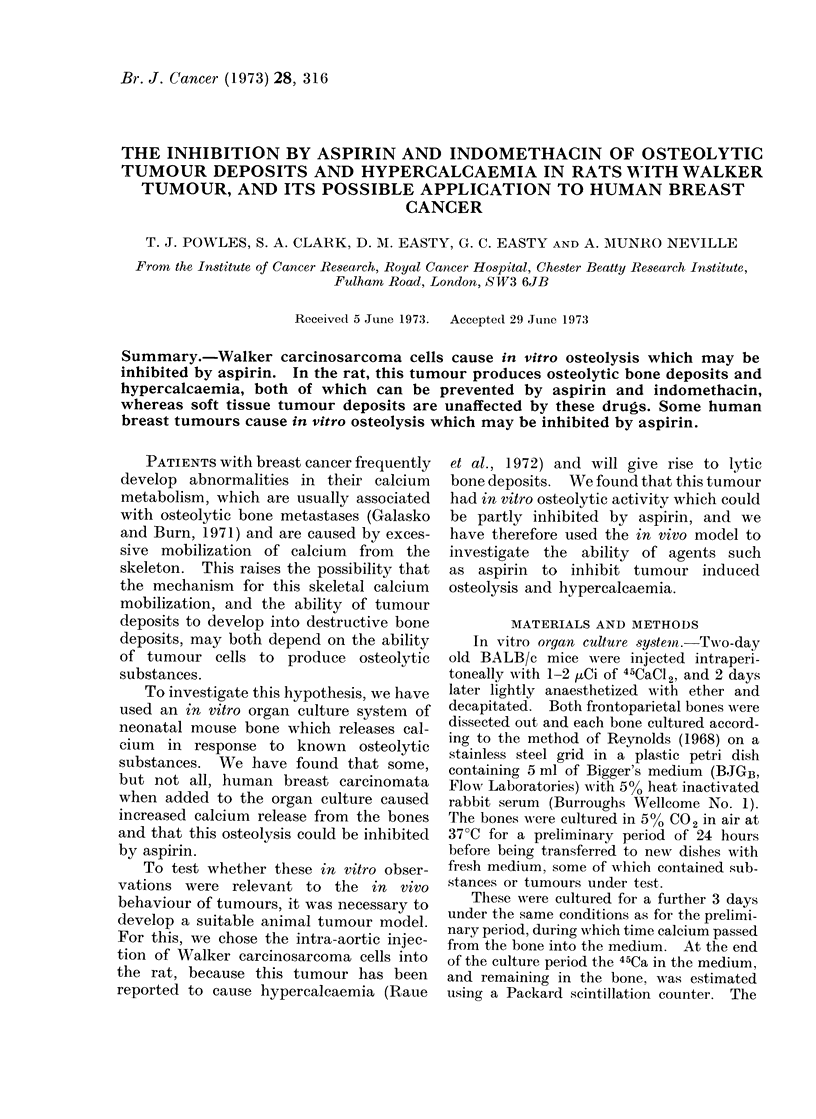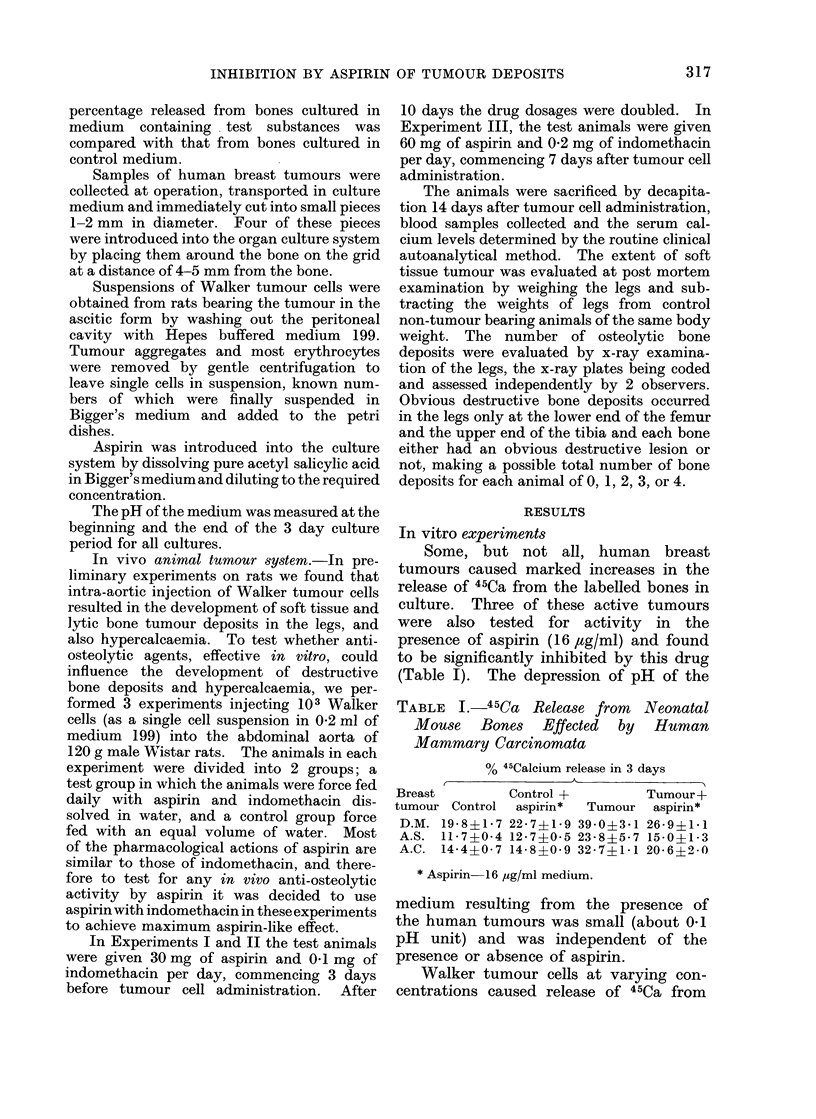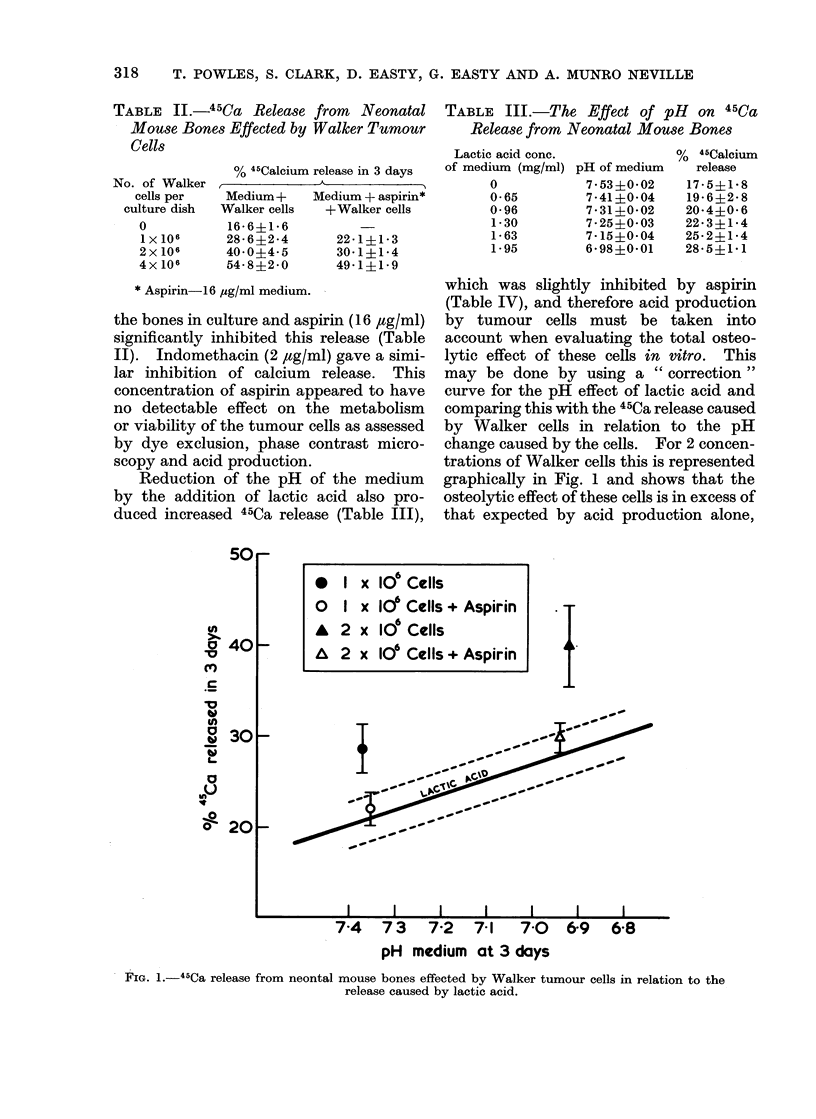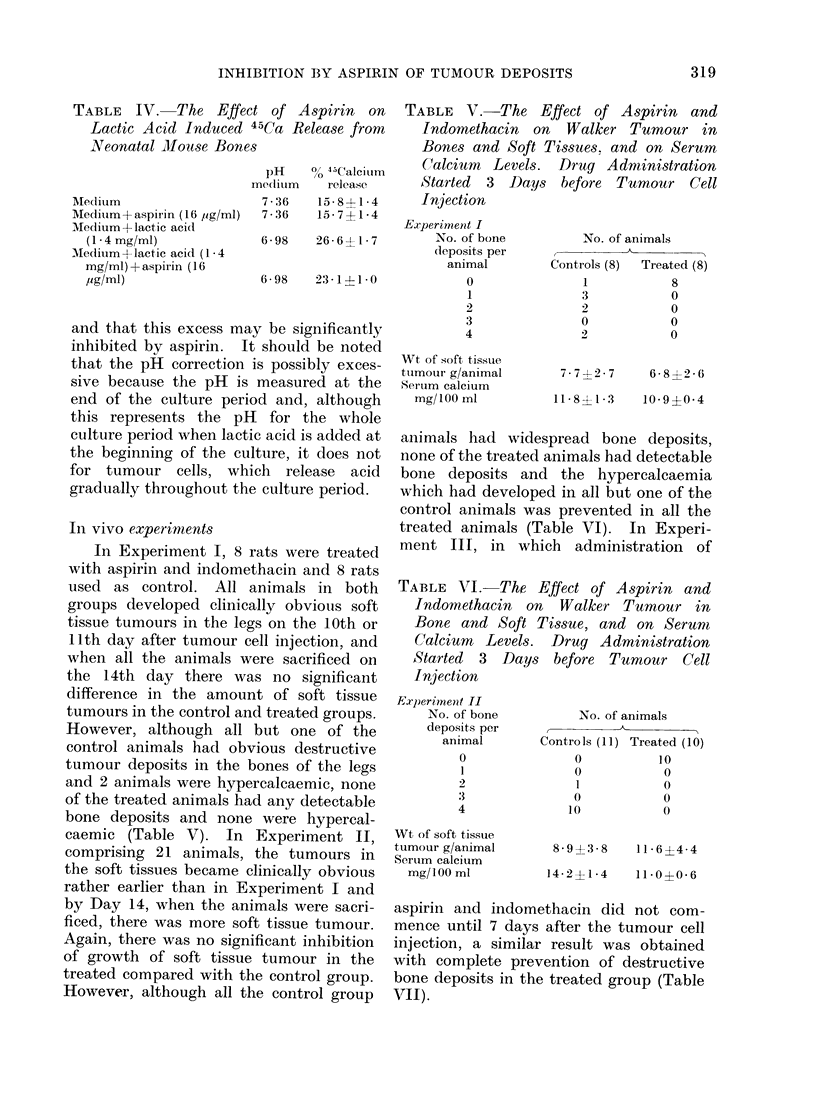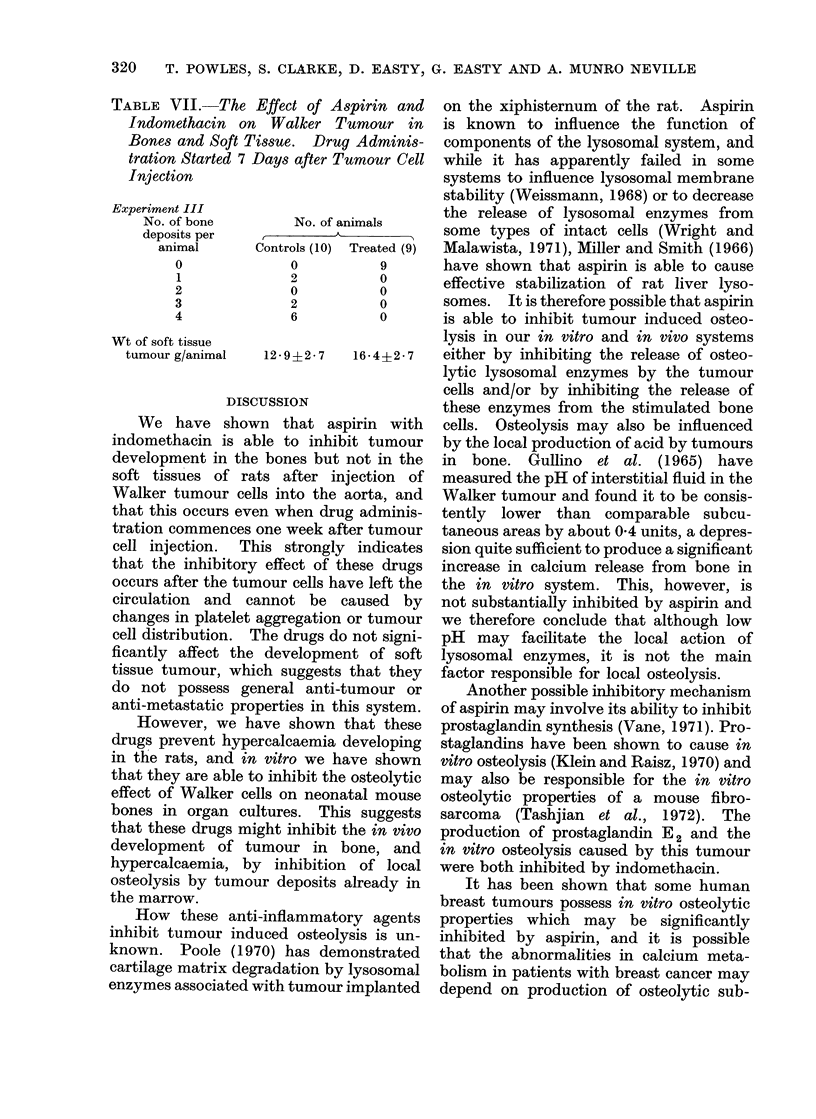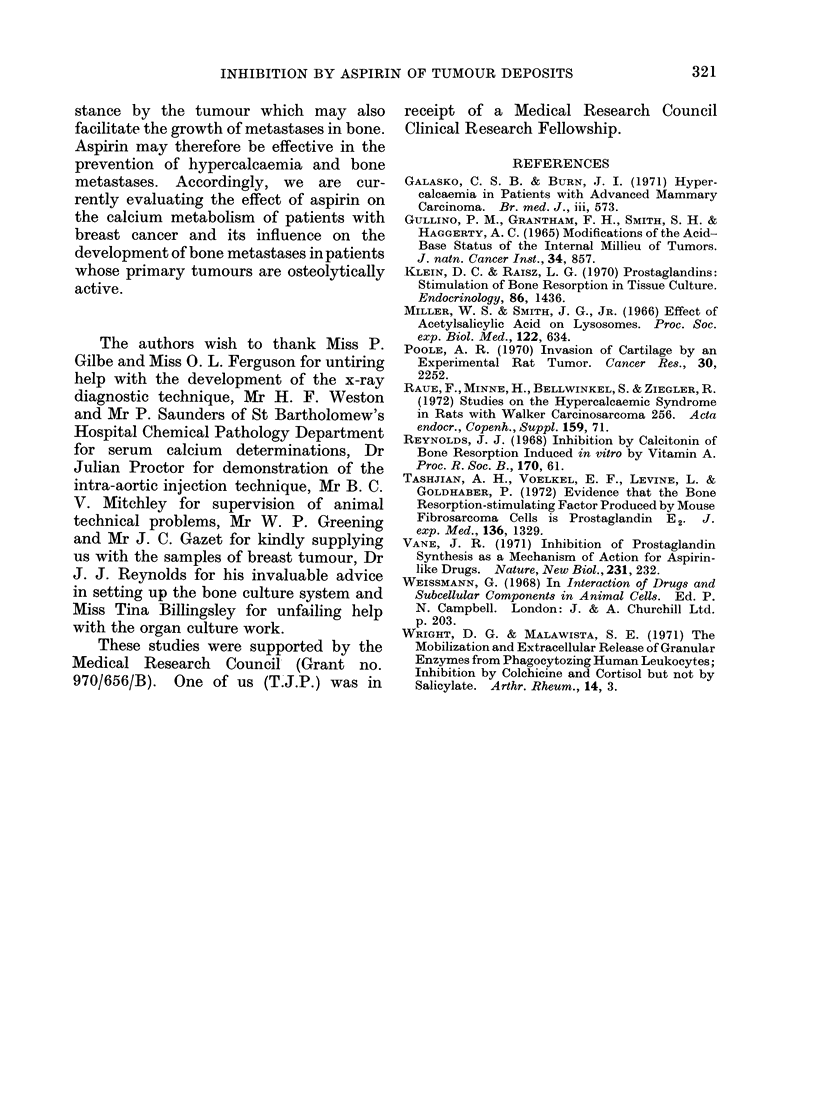# The Inhibition by Aspirin and Indomethacin of Osteolytic Tumour Deposits and Hypercalcaemia in Rats with Walker Tumour, and its Possible Application to Human Breast Cancer

**DOI:** 10.1038/bjc.1973.154

**Published:** 1973-10

**Authors:** T. J. Powles, S. A. Clark, D. M. Easty, G. C. Easty, A. Munro Neville

## Abstract

Walker carcinosarcoma cells cause *in vitro* osteolysis which may be inhibited by aspirin. In the rat, this tumour produces osteolytic bone deposits and hypercalcaemia, both of which can be prevented by aspirin and indomethacin, whereas soft tissue tumour deposits are unaffected by these drugs. Some human breast tumours cause *in vitro* osteolysis which may be inhibited by aspirin.


*Br. J. Cancer* (1973) **28**, 316

### THE INHIBITION BY ASPIRIN AND INDOMETHACIN OF OSTEOLYTIC TUMOUR DEPOSITS AND HYPERCALCAEMIA IN RATS WITH WALKER TUMOUR, AND ITS POSSIBLE APPLICATION TO HUMAN BREAST CANCER

T. J. POWLES, S. A. CLARK, D. M. EASTY, G. C. EASTY AND A. MUNRO NEVILLE

*From the Institute of Cancer Research, Royal Cancer Hospital, Chester Beatty Research Institute, Fulham Road, London, SW3 6JB*

Received 5 June 1973. Accepted 29 June 1973

**Summary.—Walker carcinosarcoma cells cause *in vitro* osteolysis which may be inhibited by aspirin. In the rat, this tumour produces osteolytic bone deposits and hypercalcaemia, both of which can be prevented by aspirin and indomethacin, whereas soft tissue tumour deposits are unaffected by these drugs. Some human breast tumours cause *in vitro* osteolysis which may be inhibited by aspirin.**

PATIENTS with breast cancer frequently develop abnormalities in their calcium metabolism, which are usually associated with osteolytic bone metastases (Galasko and Burn, 1971) and are caused by excessive mobilization of calcium from the skeleton. This raises the possibility that the mechanism for this skeletal calcium mobilization, and the ability of tumour deposits to develop into destructive bone deposits, may both depend on the ability of tumour cells to produce osteolytic substances.

To investigate this hypothesis, we have used an *in vitro* organ culture system of neonatal mouse bone which releases calcium in response to known osteolytic substances. We have found that some, but not all, human breast carcinomata when added to the organ culture caused increased calcium release from the bones and that this osteolysis could be inhibited by aspirin.

To test whether these *in vitro* observations were relevant to the *in vivo* behaviour of tumours, it was necessary to develop a suitable animal tumour model. For this, we chose the intra-aortic injection of Walker carcinosarcoma cells into the rat, because this tumour has been reported to cause hypercalcaemia (Raue *et al.*, 1972) and will give rise to lytic bone deposits. We found that this tumour had *in vitro* osteolytic activity which could be partly inhibited by aspirin, and we have therefore used the *in vivo* model to investigate the ability of agents such as aspirin to inhibit tumour induced osteolysis and hypercalcaemia.

### MATERIALS AND METHODS

In vitro *organ culture system.*—Two-day old BALB/c mice were injected intraperitoneally with 1–2 $\mu$Ci of $^{45}CaCl_2$, and 2 days later lightly anaesthetized with ether and decapitated. Both frontoparietal bones were dissected out and each bone cultured according to the method of Reynolds (1968) on a stainless steel grid in a plastic petri dish containing 5 ml of Bigger's medium ($BJG_B$, Flow Laboratories) with 5% heat inactivated rabbit serum (Burroughs Wellcome No. 1). The bones were cultured in 5% $CO_2$ in air at 37°C for a preliminary period of 24 hours before being transferred to new dishes with fresh medium, some of which contained substances or tumours under test.

These were cultured for a further 3 days under the same conditions as for the preliminary period, during which time calcium passed from the bone into the medium. At the end of the culture period the $^{45}Ca$ in the medium, and remaining in the bone, was estimated using a Packard scintillation counter. The percentage released from bones cultured in medium containing test substances was compared with that from bones cultured in control medium.

Samples of human breast tumours were collected at operation, transported in culture medium and immediately cut into small pieces 1–2 mm in diameter. Four of these pieces were introduced into the organ culture system by placing them around the bone on the grid at a distance of 4–5 mm from the bone.

Suspensions of Walker tumour cells were obtained from rats bearing the tumour in the ascitic form by washing out the peritoneal cavity with Hepes buffered medium 199. Tumour aggregates and most erythrocytes were removed by gentle centrifugation to leave single cells in suspension, known numbers of which were finally suspended in Bigger's medium and added to the petri dishes.

Aspirin was introduced into the culture system by dissolving pure acetyl salicylic acid in Bigger's medium and diluting to the required concentration.

The pH of the medium was measured at the beginning and the end of the 3 day culture period for all cultures.

In vivo *animal tumour system.*—In preliminary experiments on rats we found that intra-aortic injection of Walker tumour cells resulted in the development of soft tissue and lytic bone tumour deposits in the legs, and also hypercalcaemia. To test whether anti-osteolytic agents, effective *in vitro*, could influence the development of destructive bone deposits and hypercalcaemia, we performed 3 experiments injecting $10^3$ Walker cells (as a single cell suspension in 0·2 ml of medium 199) into the abdominal aorta of 120 g male Wistar rats. The animals in each experiment were divided into 2 groups; a test group in which the animals were force fed daily with aspirin and indomethacin dissolved in water, and a control group force fed with an equal volume of water. Most of the pharmacological actions of aspirin are similar to those of indomethacin, and therefore to test for any *in vivo* anti-osteolytic activity by aspirin it was decided to use aspirin with indomethacin in these experiments to achieve maximum aspirin-like effect.

In Experiments I and II the test animals were given 30 mg of aspirin and 0·1 mg of indomethacin per day, commencing 3 days before tumour cell administration. After 10 days the drug dosages were doubled. In Experiment III, the test animals were given 60 mg of aspirin and 0·2 mg of indomethacin per day, commencing 7 days after tumour cell administration.

The animals were sacrificed by decapitation 14 days after tumour cell administration, blood samples collected and the serum calcium levels determined by the routine clinical autoanalytical method. The extent of soft tissue tumour was evaluated at post mortem examination by weighing the legs and subtracting the weights of legs from control non-tumour bearing animals of the same body weight. The number of osteolytic bone deposits were evaluated by x-ray examination of the legs, the x-ray plates being coded and assessed independently by 2 observers. Obvious destructive bone deposits occurred in the legs only at the lower end of the femur and the upper end of the tibia and each bone either had an obvious destructive lesion or not, making a possible total number of bone deposits for each animal of 0, 1, 2, 3, or 4.

### RESULTS

### In vitro *experiments*

Some, but not all, human breast tumours caused marked increases in the release of $^{45}Ca$ from the labelled bones in culture. Three of these active tumours were also tested for activity in the presence of aspirin (16 μg/ml) and found to be significantly inhibited by this drug (Table I). The depression of pH of the medium resulting from the presence of the human tumours was small (about 0·1 pH unit) and was independent of the presence or absence of aspirin.

TABLE I.—$^{45}$*Ca Release from Neonatal Mouse Bones Effected by Human Mammary Carcinomata*

| Breast tumour | % $^{45}$Calcium release in 3 days: Control | Control + aspirin* | Tumour | Tumour + aspirin* |
|---|---|---|---|---|
| D.M. | 19·8±1·7 | 22·7±1·9 | 39·0±3·1 | 26·9±1·1 |
| A.S. | 11·7±0·4 | 12·7±0·5 | 23·8±5·7 | 15·0±1·3 |
| A.C. | 14·4±0·7 | 14·8±0·9 | 32·7±1·1 | 20·6±2·0 |

* Aspirin—16 μg/ml medium.

Walker tumour cells at varying concentrations caused release of $^{45}Ca$ from

TABLE II.—$^{45}$*Ca Release from Neonatal Mouse Bones Effected by Walker Tumour Cells*

| No. of Walker cells per culture dish | % $^{45}$Calcium release in 3 days: Medium + Walker cells | Medium + aspirin* + Walker cells |
|---|---|---|
| 0 | 16·6±1·6 | — |
| 1×10$^6$ | 28·6±2·4 | 22·1±1·3 |
| 2×10$^6$ | 40·0±4·5 | 30·1±1·4 |
| 4×10$^6$ | 54·8±2·0 | 49·1±1·9 |

* Aspirin—16 μg/ml medium.

the bones in culture and aspirin (16 μg/ml) significantly inhibited this release (Table II). Indomethacin (2 μg/ml) gave a similar inhibition of calcium release. This concentration of aspirin appeared to have no detectable effect on the metabolism or viability of the tumour cells as assessed by dye exclusion, phase contrast microscopy and acid production.

Reduction of the pH of the medium by the addition of lactic acid also produced increased $^{45}$Ca release (Table III),

TABLE III.—*The Effect of pH on* $^{45}$*Ca Release from Neonatal Mouse Bones*

| Lactic acid conc. of medium (mg/ml) | pH of medium | % $^{45}$Calcium release |
|---|---|---|
| 0 | 7·53±0·02 | 17·5±1·8 |
| 0·65 | 7·41±0·04 | 19·6±2·8 |
| 0·96 | 7·31±0·02 | 20·4±0·6 |
| 1·30 | 7·25±0·03 | 22·3±1·4 |
| 1·63 | 7·15±0·04 | 25·2±1·4 |
| 1·95 | 6·98±0·01 | 28·5±1·1 |

which was slightly inhibited by aspirin (Table IV), and therefore acid production by tumour cells must be taken into account when evaluating the total osteolytic effect of these cells *in vitro*. This may be done by using a "correction" curve for the pH effect of lactic acid and comparing this with the $^{45}$Ca release caused by Walker cells in relation to the pH change caused by the cells. For 2 concentrations of Walker cells this is represented graphically in Fig. 1 and shows that the osteolytic effect of these cells is in excess of that expected by acid production alone,

FIG. 1.—$^{45}$Ca release from neontal mouse bones effected by Walker tumour cells in relation to the release caused by lactic acid.

TABLE IV.—*The Effect of Aspirin on Lactic Acid Induced $^{45}$Ca Release from Neonatal Mouse Bones*

| | pH medium | % $^{45}$Calcium release |
|---|---|---|
| Medium | 7·36 | 15·8±1·4 |
| Medium+aspirin (16 μg/ml) | 7·36 | 15·7±1·4 |
| Medium+lactic acid (1·4 mg/ml) | 6·98 | 26·6±1·7 |
| Medium+lactic acid (1·4 mg/ml)+aspirin (16 μg/ml) | 6·98 | 23·1±1·0 |

and that this excess may be significantly inhibited by aspirin. It should be noted that the pH correction is possibly excessive because the pH is measured at the end of the culture period and, although this represents the pH for the whole culture period when lactic acid is added at the beginning of the culture, it does not for tumour cells, which release acid gradually throughout the culture period.

In vivo *experiments*

In Experiment I, 8 rats were treated with aspirin and indomethacin and 8 rats used as control. All animals in both groups developed clinically obvious soft tissue tumours in the legs on the 10th or 11th day after tumour cell injection, and when all the animals were sacrificed on the 14th day there was no significant difference in the amount of soft tissue tumours in the control and treated groups. However, although all but one of the control animals had obvious destructive tumour deposits in the bones of the legs and 2 animals were hypercalcaemic, none of the treated animals had any detectable bone deposits and none were hypercalcaemic (Table V). In Experiment II, comprising 21 animals, the tumours in the soft tissues became clinically obvious rather earlier than in Experiment I and by Day 14, when the animals were sacrificed, there was more soft tissue tumour. Again, there was no significant inhibition of growth of soft tissue tumour in the treated compared with the control group. However, although all the control group

TABLE V.—*The Effect of Aspirin and Indomethacin on Walker Tumour in Bones and Soft Tissues, and on Serum Calcium Levels. Drug Administration Started 3 Days before Tumour Cell Injection*

*Experiment I*

| No. of bone deposits per animal | No. of animals | |
|---|---|---|
| | Controls (8) | Treated (8) |
| 0 | 1 | 8 |
| 1 | 3 | 0 |
| 2 | 2 | 0 |
| 3 | 0 | 0 |
| 4 | 2 | 0 |
| Wt of soft tissue tumour g/animal | 7·7±2·7 | 6·8±2·6 |
| Serum calcium mg/100 ml | 11·8±1·3 | 10·9±0·4 |

animals had widespread bone deposits, none of the treated animals had detectable bone deposits and the hypercalcaemia which had developed in all but one of the control animals was prevented in all the treated animals (Table VI). In Experiment III, in which administration of aspirin and indomethacin did not commence until 7 days after the tumour cell injection, a similar result was obtained with complete prevention of destructive bone deposits in the treated group (Table VII).

TABLE VI.—*The Effect of Aspirin and Indomethacin on Walker Tumour in Bone and Soft Tissue, and on Serum Calcium Levels. Drug Administration Started 3 Days before Tumour Cell Injection*

*Experiment II*

| No. of bone deposits per animal | No. of animals | |
|---|---|---|
| | Controls (11) | Treated (10) |
| 0 | 0 | 10 |
| 1 | 0 | 0 |
| 2 | 1 | 0 |
| 3 | 0 | 0 |
| 4 | 10 | 0 |
| Wt of soft tissue tumour g/animal | 8·9±3·8 | 11·6±4·4 |
| Serum calcium mg/100 ml | 14·2±1·4 | 11·0±0·6 |

TABLE VII.—*The Effect of Aspirin and Indomethacin on Walker Tumour in Bones and Soft Tissue. Drug Administration Started 7 Days after Tumour Cell Injection*

*Experiment III*

| No. of bone deposits per animal | No. of animals Controls (10) | Treated (9) |
|---|---|---|
| 0 | 0 | 9 |
| 1 | 2 | 0 |
| 2 | 0 | 0 |
| 3 | 2 | 0 |
| 4 | 6 | 0 |
| Wt of soft tissue tumour g/animal | 12·9±2·7 | 16·4±2·7 |

### DISCUSSION

We have shown that aspirin with indomethacin is able to inhibit tumour development in the bones but not in the soft tissues of rats after injection of Walker tumour cells into the aorta, and that this occurs even when drug administration commences one week after tumour cell injection. This strongly indicates that the inhibitory effect of these drugs occurs after the tumour cells have left the circulation and cannot be caused by changes in platelet aggregation or tumour cell distribution. The drugs do not significantly affect the development of soft tissue tumour, which suggests that they do not possess general anti-tumour or anti-metastatic properties in this system.

However, we have shown that these drugs prevent hypercalcaemia developing in the rats, and *in vitro* we have shown that they are able to inhibit the osteolytic effect of Walker cells on neonatal mouse bones in organ cultures. This suggests that these drugs might inhibit the *in vivo* development of tumour in bone, and hypercalcaemia, by inhibition of local osteolysis by tumour deposits already in the marrow.

How these anti-inflammatory agents inhibit tumour induced osteolysis is unknown. Poole (1970) has demonstrated cartilage matrix degradation by lysosomal enzymes associated with tumour implanted on the xiphisternum of the rat. Aspirin is known to influence the function of components of the lysosomal system, and while it has apparently failed in some systems to influence lysosomal membrane stability (Weissmann, 1968) or to decrease the release of lysosomal enzymes from some types of intact cells (Wright and Malawista, 1971), Miller and Smith (1966) have shown that aspirin is able to cause effective stabilization of rat liver lysosomes. It is therefore possible that aspirin is able to inhibit tumour induced osteolysis in our *in vitro* and *in vivo* systems either by inhibiting the release of osteolytic lysosomal enzymes by the tumour cells and/or by inhibiting the release of these enzymes from the stimulated bone cells. Osteolysis may also be influenced by the local production of acid by tumours in bone. Gullino *et al.* (1965) have measured the pH of interstitial fluid in the Walker tumour and found it to be consistently lower than comparable subcutaneous areas by about 0·4 units, a depression quite sufficient to produce a significant increase in calcium release from bone in the *in vitro* system. This, however, is not substantially inhibited by aspirin and we therefore conclude that although low pH may facilitate the local action of lysosomal enzymes, it is not the main factor responsible for local osteolysis.

Another possible inhibitory mechanism of aspirin may involve its ability to inhibit prostaglandin synthesis (Vane, 1971). Prostaglandins have been shown to cause *in vitro* osteolysis (Klein and Raisz, 1970) and may also be responsible for the *in vitro* osteolytic properties of a mouse fibrosarcoma (Tashjian *et al.*, 1972). The production of prostaglandin $E_2$ and the *in vitro* osteolysis caused by this tumour were both inhibited by indomethacin.

It has been shown that some human breast tumours possess *in vitro* osteolytic properties which may be significantly inhibited by aspirin, and it is possible that the abnormalities in calcium metabolism in patients with breast cancer may depend on production of osteolytic substance by the tumour which may also facilitate the growth of metastases in bone. Aspirin may therefore be effective in the prevention of hypercalcaemia and bone metastases. Accordingly, we are currently evaluating the effect of aspirin on the calcium metabolism of patients with breast cancer and its influence on the development of bone metastases in patients whose primary tumours are osteolytically active.

The authors wish to thank Miss P. Gilbe and Miss O. L. Ferguson for untiring help with the development of the x-ray diagnostic technique, Mr H. F. Weston and Mr P. Saunders of St Bartholomew's Hospital Chemical Pathology Department for serum calcium determinations, Dr Julian Proctor for demonstration of the intra-aortic injection technique, Mr B. C. V. Mitchley for supervision of animal technical problems, Mr W. P. Greening and Mr J. C. Gazet for kindly supplying us with the samples of breast tumour, Dr J. J. Reynolds for his invaluable advice in setting up the bone culture system and Miss Tina Billingsley for unfailing help with the organ culture work.

These studies were supported by the Medical Research Council (Grant no. 970/656/B). One of us (T.J.P.) was in receipt of a Medical Research Council Clinical Research Fellowship.

### REFERENCES

GALASKO, C. S. B. & BURN, J. I. (1971) Hypercalcaemia in Patients with Advanced Mammary Carcinoma. *Br. med. J.*, iii, 573.

GULLINO, P. M., GRANTHAM, F. H., SMITH, S. H. & HAGGERTY, A. C. (1965) Modifications of the Acid–Base Status of the Internal Millieu of Tumors. *J. natn. Cancer Inst.*, **34**, 857.

KLEIN, D. C. & RAISZ, L. G. (1970) Prostaglandins: Stimulation of Bone Resorption in Tissue Culture. *Endocrinology*, **86**, 1436.

MILLER, W. S. & SMITH, J. G., JR. (1966) Effect of Acetylsalicylic Acid on Lysosomes. *Proc. Soc. exp. Biol. Med.*, **122**, 634.

POOLE, A. R. (1970) Invasion of Cartilage by an Experimental Rat Tumor. *Cancer Res.*, **30**, 2252.

RAUE, F., MINNE, H., BELLWINKEL, S. & ZIEGLER, R. (1972) Studies on the Hypercalcaemic Syndrome in Rats with Walker Carcinosarcoma 256. *Acta endocr., Copenh., Suppl.* **159**, 71.

REYNOLDS, J. J. (1968) Inhibition by Calcitonin of Bone Resorption Induced *in vitro* by Vitamin A. *Proc. R. Soc. B.*, **170**, 61.

TASHJIAN, A. H., VOELKEL, E. F., LEVINE, L. & GOLDHABER, P. (1972) Evidence that the Bone Resorption-stimulating Factor Produced by Mouse Fibrosarcoma Cells is Prostaglandin $E_2$. *J. exp. Med.*, **136**, 1329.

VANE, J. R. (1971) Inhibition of Prostaglandin Synthesis as a Mechanism of Action for Aspirin-like Drugs. *Nature, New Biol.*, **231**, 232.

WEISSMANN, G. (1968) In *Interaction of Drugs and Subcellular Components in Animal Cells.* Ed. P. N. Campbell. London: J. & A. Churchill Ltd. p. 203.

WRIGHT, D. G. & MALAWISTA, S. E. (1971) The Mobilization and Extracellular Release of Granular Enzymes from Phagocytozing Human Leukocytes; Inhibition by Colchicine and Cortisol but not by Salicylate. *Arthr. Rheum.*, **14**, 3.